# A humanized anti-CD26 monoclonal antibody inhibits cell growth of malignant mesothelioma via retarded G2/M cell cycle transition

**DOI:** 10.1186/s12935-016-0310-9

**Published:** 2016-04-30

**Authors:** Mutsumi Hayashi, Hiroko Madokoro, Koji Yamada, Hiroko Nishida, Chikao Morimoto, Michiie Sakamoto, Taketo Yamada

**Affiliations:** Department of Pathology, Keio University School of Medicine, 35 Shinanomachi, Tokyo, Shinjuku-ku 160-8582 Japan; Department of Pediatrics, Keio University School of Medicine, 35 Shinanomachi, Tokyo, Shinjuku-ku 160-8582 Japan; Laboratory of Nuclear Transport Dynamics, National Insititute of Biomedical Innovation, 7-6-8 Saito-Asagi, Ibaraki City, Osaka Prefecture 567-0085 Japan; Department of Therapy Development and Innovation for Immune Disorders and Cancers, Juntendo University, 2-1-2 Hongo, Tokyo, Bunkyo-ku 113-8421 Japan; Department of Pathology, Saitama Medical University, 38 Morohongo, Saitama, Moroyama-machi 350-0495 Japan

**Keywords:** Mesothelioma, CD26, Monoclonal antibody, G2/M transition, Pemetrexed

## Abstract

**Background:**

Malignant Mesothelioma (MM) is a highly aggressive tumor with poor prognosis. Multimodal treatments and novel molecular targeted therapies against MM are in high demand in order treat this disease effectively. We have developed a humanized monoclonal antibody YS110 against CD26 expressed in 85 % of MM cases. CD26 is thought to be involved in tumor growth and invasion by interacting with collagen and fibronectin, or affecting signal transduction processes.

**Methods:**

We evaluated the direct anti-tumor effect of YS110 against MM cell lines, NCI-H2452 and JMN, and investigated its effects on cell cycle and on the cell cycle regulator molecules. In addition, we investigated synergistic effects of YS110 and anti-tumor agent pemetrexed (PMX) against MM cell line both in vitro and in vivo.

**Results:**

YS110 suppressed the proliferation of NCI-H2452 cells by approximately 20 % in 48 h. Based on cell cycle analysis, percentage of cells in G2/M phase increased 8.0 % on the average after YS110 treatment; in addition, cell cycle regulator p21 cip/waf1 was increased and cyclin B1 was decreased after YS110 treatment. Inhibitory phosphorylation of both cdc2 (Tyr15) and cdc25C (Ser216) were elevated. Furthermore, activating phosphorylation of p38 MAPK (Thr180/Tyr182) and ERK1/2 (Thr202/Tyr204) were augmented at 24 h after YS110 treatment. PMX rapidly induced CD26 expression on cell surface and the treatment with both YS110 and PMX inhibited in vivo tumor growth accompanied by a synergistic reduction in the MIB-1 index.

**Conclusion:**

This is a first report of a novel anti-proliferative mechanism of the humanized anti-CD26 monoclonal antibody YS110, which resulted in G2/M cell cycle delay through regulation of quantity and activity of various cell cycle regulating molecules.

## Background

Malignant mesothelioma (MM) is an aggressive cancer of the pleura, peritoneal cavity, pericardium, and scrotum and has a poor prognosis. MM is associated with occupational exposure to asbestos and, despite legislation introduced by many industrialized countries, the incidence is not expected to peak until 2020 due to the long latency between initial exposure and disease expression [1]. As single modality approach to treatment has failed to extend survival, multimodal treatment and novel molecular targeted therapies are highly sought after. Although extrapleural pneumonectomy (EPP) is a preferred treatment option, median survival among patients receiving EPP alone is less than 10 months [2]. EPP followed by high-dose radiation therapy (RT) has been shown to prolong median survival to 33.8 months in patients with Stage 1 and Stage 2 MM but survival remained 10 months in patients with Stage 3 and Stage 4 MM [2]. A Phase 3 trial showed that combination of pemetrexed and cisplatin improved survival over cisplatin alone for inoperable patients [3]. According to recent multicenter trials of trimodality treatment that consisted of neoadjuvant chemotherapy (cisplatin and pemetrexed), EPP, and adjuvant RT led in the USA [2] and Europe [4], median survival of patients who completed the therapy was 29.1 months compared to 18.4 months in controls. Since the trimodality approach seems to be limited and because not all patient can tolerate aggressive therapies, novel molecular targeted therapies are highly desirable. To date, a number of molecular targeted agents have been evaluated in MM. While tyrosine kinase inhibitors against epidermal growth factor receptor (EGFR) and platelet-derived growth factor receptor (PDGFR) did not show clinically significant effects, histone deacetylase inhibitor (HDACI) and anti-angiogenic agents showed some clinical benefits and are undergoing Phase 3 trials [5]; however, none of these agents have been incorporated into clinical practice and efforts must continue in the area of both clinical research and search for novel target molecules.

CD26 is an 110 kD glycoprotein anchored in the cellular membrane with dipeptidyl peptidase IV activity. CD26 is also known as a co-stimulatory molecule of the T lymphocyte. CD26 binds to caveolin-1 on antigen-presenting cells and the interaction triggers signal transduction process leading to T cell proliferation and cytokine production [6]. Several recent studies have shown that CD26 is highly expressed in several malignancies, including MM, lung adenocarcinoma, hepatocellular carcinoma, prostate cancer, and thyroid cancer [7]. CD26 expression evaluated by immunohistochemistry was positive in 85 % of tested MM cases [8, 9]. Moreover, CD26 is thought to be involved in tumor growth and invasion through its interaction with collagen and fibronectin or by regulating activity of chemotactic peptides through its DPPIV activity. Furthermore, CD26 has been reported to be involved in signal transduction processes, including the p38 MAPK pathway. Though the mechanisms of action of CD26 have not been clarified, its enzymatic activity does not appear essential for its role in signal transduction process [10]. Considering its high rate of overexpression in MM and suspected function in tumor progression, we have developed a humanized an anti-CD26 antibody, designated YS110, as a targeted therapy against CD26-positive malignancies, including MM. We have previously reported the anti-tumor effects of YS110 against MM cells [11]. In addition to the anti-tumor effect via antibody-dependent-cell-mediated-cytotoxicity, YS110 showed direct anti-tumor effect via p27^kip1^ accumulation [11]; however, the molecular mechanism of direct anti-tumor effect of YS110 against MM cell lines remains unknown.

The molecular mechanisms underlying the direct anti-tumor effect of several monoclonal antibodies have been investigated; for example, the anti-HER-2 antibody (Trastuzumab) and anti-EGFR antibody (Cetuximab) result in G1/S cell cycle arrest by upregulating the CDK inhibitor p27^kip1^ via multiple signaling pathways [12, 13]. The anti-CD20 antibody (rituximab) can induce cell death of malignant B cell lymphoma cells in vitro via inhibition of the p38 MAPK, ERK1/2, and AKT anti-apoptotic survival pathways [14]. Most therapeutic antibodies against cancers that affect the cell cycle, including antibodies mentioned above, result in G1/S arrest. So far, only one anti-cancer antibody, the anti-human type 1 insulin-like growth factor receptor (IGF-IR) antibody A12 against androgen-independent prostate cancer cell line LuCaP 35 V, has been reported to cause G2/M cell cycle delay although its molecular mechanism is not yet understood [15].

In this study, we focused on evaluating the direct in vitro effect of the humanized anti-CD26 monoclonal antibody YS110 against the MM cell line NCI-H2452 and investigated its effect on the cell cycle and on cell cycle-regulating molecules. YS110 inhibited growth of YS110 with G2/M cell cycle arrest and altered the expression or phosphorylation state of cell cycle molecules. Furthermore, pemetrexed (PMX), a standard reagent against mesothelioma, rapidly induced CD26 expression on the cell surface and treatment with both YS110 and PMX inhibited in vivo tumor growth in a synergistic manner. This is the first report describing a novel anti-proliferative mechanism of the humanized anit-CD26 monoclonal antibody YS110, which resulted in G2/M cell cycle delay, through regulation of quantity and activity of various cell cycle-regulating molecules.

## Results

### YS110 inhibits mesothelioma cell proliferation

**Fig. 1:**
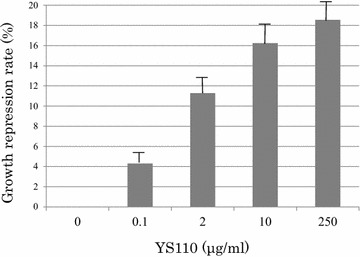
YS110 inhibits NCI-H2452 cell proliferation. WST-1 assay revealed inhibition of NCI-H2452 cell proliferation with YS110 treatment in a concentration-dependent manner up to 18.6 %. The experiment was performed using 96 multi-well plates

YS110 inhibits proliferation of the mesothelioma cell line NCI-H2452 in a concentration-dependent manner (Fig. 1). Maximum of growth inhibition was 18.3 % at 250 μg/mL of YS110. Based on this result, we used YS110 at 2 μg/mL, which showed 11.2 % of growth inhibition, in the following experiments.

### YS110 induced G2/M cell cycle delay in mesothelioma cells

**Fig. 2:**
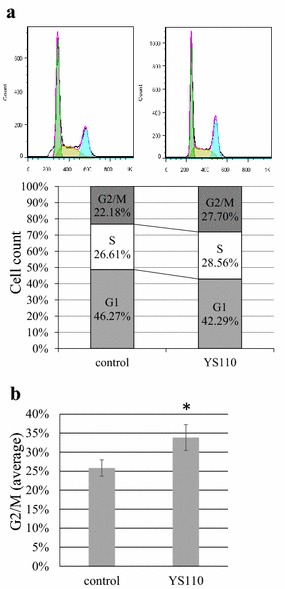
YS110 induces G2/M cell cycle delay in NCI-H2452 cells. **a** Flowcytometry analysis using propidium iodide staining revealed that the percentage of G2/M phase cells increased while the percentage of G1 phase cells was reduced in NCI-H2452 cells treated 24 h with 2 μg/mL of YS110. A representative experiment is shown. **b** The average percentage of G2/M phase cells in NCI-H2452 cells treated 24 h with 2 μg/mL of YS110 was significantly increased (*p < 0.05)

To investigate the mechanism responsible for the growth inhibition caused by YS110, cell-cycle distribution was determined by flow cytometry analysis. At 24 h after YS110 treatment, the percentage of cells in the G2/M phase increased compared to the control. The representative experiment is shown (Fig. 2a). On the average of ten experiments, the percentage of G2/M phase cells were significantly increased after YS110 treatment (p < 0.05) (Fig. 2b). This cell cycle delay may be compatible with repression of cell proliferation. Furthermore, in another CD26 positive MM cell line NCI-H28, the percentage of cells in G2/M phase increased by 5 % on the average after YS110 treatment though its significance could not be proved statistically (data not shown).

### YS110 alters cell cycle regulators

**Fig. 3:**
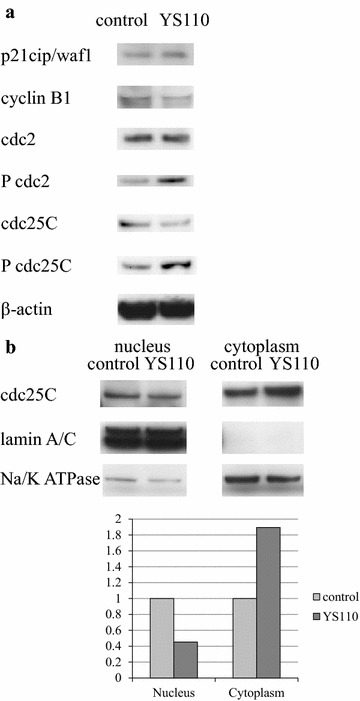
YS110 alters expressions and phosphorylation of cell cycle regulators. **a** At 24 h after treatment with 2 μg/mL of YS110, p21 protein expression increased while cyclinB1 protein expression decreased. Inhibitory phosphorylation of cdc2 Tyr15 and cdc25C Ser216 were elevated while there was no significant change in whole cdc2 and cdc25C protein. β-actin was used as an internal control. **b** Alteration in subcellular distribution of whole cdc25C by YS110 treatment is shown. At 24 h after treatment with 2 μg/mL of YS110, nuclear cdc25C protein decreased while cytoplasmic cdc25C protein increased. LaminA/C was used as a nuclear marker and Na–K ATPase was used as a cytoplasmic marker. The *bar graph* demonstrates the densitometric analysis of a Western blot of whole cdc25C. Results are normalized by the densitometry of control cells. All experiments were performed in triplicate and a representative experiment is shown

We investigated the alterations caused by YS110 treatment in the quantity and activation state of cell cycle regulators responsible for G2/M transition. At 24 h after YS110 treatment, the cell cycle regulator p21 increased while the positive regulatory subunit cyclin B1 decreased. Inhibitory phosphorylation of cdc2 on Tyr15 and inhibitory phosphorylation of cdc25C on Ser216, an upstream inhibitory regulator of cdc2, was elevated (Fig. 3a). Cdc25C phosphorylated on Ser216 is known to be sequestrated into cytoplasm and refrained from contact with cdc2 [16]. After YS110 treatment for 24 h, cytoplasmic whole cdc25C was elevated while nuclear whole cdc25C was decreased, as confirmed by densitometry analysis (Fig. 3b). At 6 h and 12 h after YS110 treatment, the amount of phosphorylated cdc2 and phosphorylated cdc25C were varied among experiments despite consistent increase at 24 h. No significant change in cdc25A and cdc25B was observed (data not shown).

### YS110 elevates activating phosphorylation of p38 MAPK and ERK1/2

**Fig. 4:**
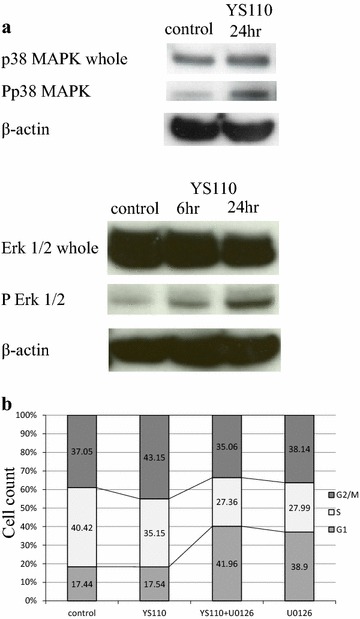
YS110 augments phosphorylation of p38MAPK and ERK1/2. **a** Activating phosohorylation of p38 (Thr180/Tyr182) and ERK1/2 (Thr202/Tyr204) were elevated by 24 h after treatment with YS110 while no substantial changes of whole p38 and ERK1/2 protein were observed. β-actin was used as an internal control. **b** G2/M arrest caused by YS110 treatment was reversed by ERK1/2 activation inhibitor U0126 (10 μM). A proportion (43.15 %) of G2/M was elevated in cells with YS110 treatment compared with in cells without YS110 (control, 37.05 %) and the proportion of G2/M decreased in cells with both YS110 and U0126 treatment (35.06 %). All experiments were performed in triplicate and a representative experiment is shown

In order to determine the upstream regulator of cdc25C phosphorylation caused by YS110 treatment, expression and activation status of several molecules known to regulate cell cycle through cdc25C phosphorylation were examined. Activating phosphorylation of p38 MAPK (Thr180/Tyr182) and ERK1/2 (Thr202/Tyr204) were elevated 24 h after YS110 treatment (Fig. 4a). No significant change in chk1, chk2, or c-TAK1 was observed (data not shown). While the p38 inhibitor SB203580 failed to block G2/M arrest caused by YS110 (data not shown), the MEK1/2 inhibitor U0126 blocked G2/M arrest caused by YS110 according to cell cycle analysis using flowcytometry (Fig. 4b).

### *Pemetrexed (PMX) increased CD26 expression in mesothelioma cells* in vitro

**Fig. 5:**
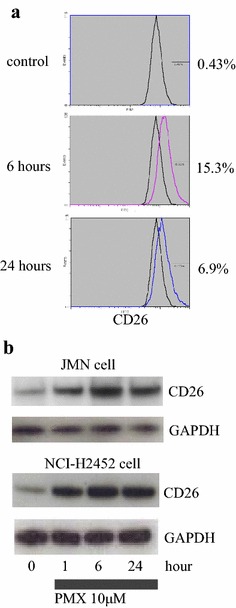
Pemetrexed (PMX) increased CD26 expression in vitro. **a** Based on flowcytometry analysis, cell surface CD26 expression on JMN cells increased 6-24 h after treatment with 10 μM of PMX. **b** Based on Western blot analysis, the expression of CD26 protein was rapidly induced in whole cell lysates by treatment with 10 μM of PMX at 1–24 h. GAPDH was used as an internal control. All experiments were performed in triplicate and a representative experiment is shown

CD26 expression on the cell surface of JMN cells increased 15 % from 6 to 6.5 % 24 h after treatment with 10 μM of PMX based on flowcytometry analysis (Fig. 5a). In order to confirm the augmented expression of CD26 in JMN cells, Western blot analysis was performed. CD26 protein expression was rapidly induced in whole cell lysates by treatment with 10 μM of PMX at 1 h after PMX treatment; most augmentation of CD26 expression at 6 h and then this augmented expression continued to 24 h after PMX treatment (Fig. 5b). In order to examine the altered expression of CD26 in NCI-H2452 cells, Western blot analysis was performed. CD26 protein expression in NCI-H2452 cells was also rapidly induced in whole cell lysates by treatment with 10 μM of PMX at 1 h after PMX treatment; most augmentation of CD26 expression at 6 h and then this augmented expression continued to 24 h after PMX treatment (Fig. 5b).

### *Effective inhibition of* in vivo *mesothelioma cell growth by combined treatment with both YS110 and PMX*

**Fig. 6:**
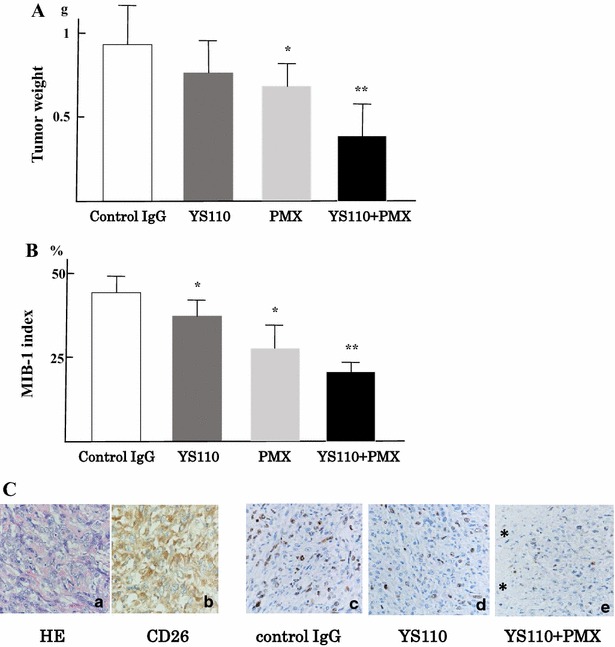
Effective inhibition of mesothelioma cell growth in vivo. **A** Combination effects of YS110 and PMX were examined using xenograft models with JMN cells transplantation subcutaneously. The weight of tumors treated with YS110 was insignificantly reduced but PMX treatment induced a significantly reduced tumor weight (*p < 0.05). A combination of YS110 and PMX treatment reduced tumor weight compared with the weight of tumors with YS110 single treatment and PMX single treatment synergistically (**p < 0.05). **B** Combination effects of tumor growth in vivo were examined using a measurement of MIB-1 index histologically. MIB-1 index was significantly reduced with YS110 or PMX single treatment compared with control IgG treatment (*p < 0.05). The MIB-1 index after combinatory treatment with YS110 and PMX was significantly reduced compared with YS110 or PMX single treatment (**p < 0.05). **C** Sarcomatous mesothelioma is shown in HE staining (*a*) and stained with anti-CD26 polyclonal antibody (R&D) (*b*) MIB-1 index was measured by immunohistochemistry using the anti-Ki-67 monoclonal antibody (DAKO, clone MIB-1). Staining of Ki-67 antigens in nucleus is seen in tumors treated with control IgG (*c*), YS110 (*d*), and both YS110 and PMX (*e*). The number of Ki-67 positive nucleus was reduced after combinatory treatment of YS110 and PMX and the necrotic area is indicated by an *asterisk* (*e*)

Combination effects of YS110 and PMX were examined using xenograft models with JMN cells transplanted into NOG mice subcutaneously. Mice were then monitored for the development and progression of tumors and the tumor size was determined by caliper measurement. Tumor size in mice treated with both YS110 and PMX was smaller than mice treated with only YS110 or PMX (data not shown). The weight of tumors with YS110 treatment was insignificantly reduced (Fig. 6A). PMX treatment induced a significant reduction in tumor weight (*p* < 0.05); the combination of YS110 and PMX treatment synergistically reduced tumor weight compared with YS110 single treatment and PMX single treatment (*p* < 0.05).

Combination effects of tumor growth in vivo were examined by measuring the MIB-1 index histologically. The MIB-1 index was significantly decreased in tumors after YS110 or PMX single treatment compared with controls (*p* < 0.05; Fig. 6B, C). Combinatory treatment with YS110 and PMX significantly reduced the MIB-1 index compared to single treatment (*p* < 0.05; Fig. 6B, C).

Histology of the tumor derived from JMN cells in the xenograft model is shown in Fig. 6C. Sarcomatous mesothelioma is shown in HE staining (Fig. 6C-a) and stained with anti-CD26 polyclonal antibody (R&D) (Fig. 6C-b). The MIB-1 index was measured by immunohistochemistry using the anti-Ki-67 (MIB-1) monoclonal antibody. Staining of Ki-67 antigens in nucleus was shown in tumors treated with control IgG (Fig. 6C-c), YS110 (Fig. 6C-d), and both YS110 and PMX (Fig. 6C-e). The number of Ki-67 positive nuclei was decreased after combined treatment of YS110 and PMX and the necrotic area is indicated by an asterisk (Fig. 6C-e).

## Discussion

Novel molecular targeted therapies are in high demand since the aggressive trimodality approach against MM has been proved to be limited. We developed a humanized monoclonal antibody against CD26, designated as YS110, a molecular targeted therapy against MM [11]. We expected YS110 to cause ADCC to eliminate CD26 positive MM cells. In addition to the ADCC effect, YS110 demonstrated direct anti-proliferative effects in vitro against CD26-positive MM cell lines. We proposed that investigation into the molecular mechanisms of the direct anti-proliferative effect against MM cells would be beneficial for both understanding anti-tumor effect of YS110, as well as uncover the underlying mechanism its proliferation and progression. Although the direct anti-proliferative effect of YS110 is limited to a repression rate of approximately 20 % in vitro, the molecular mechanism was associated with the proliferative signal transduction system and cell cycle. Cell cycle analysis revealed that YS110 caused significant delay of the G2/M transition. To our knowledge, only one anti-cancer MoAb, anti-IGF-IR antibody A12 against the androgen-independent prostate cancer cell line LaCaP 35 V, has been reported to result in G2/M delay although its molecular mechanism is not yet understood [15]. Therefore, we are the first to report results on the molecular mechanisms underlying G2/M delay from a cancer targeted-antibody. In order to elucidate the mechanism of G2/M delay mediated by YS110, we analyzed the quantities and phosphorylation status of G2/M regulators using Western blot analysis. At 24 h after treatment with YS110, inhibitory phosphorylation of cdc2 and its upstream regulator cdc25C were elevated. Phosphorylation of Cdc25C on Ser216 is sequestrated into the cytoplasm and therefore restrained from contact with nuclear cdc2. Increased levels of cytoplasmic whole cdc25 protein and decreased levels of nuclear whole cdc25C protein at 24 h occurred after YS110 treatment, confirming sequestration. Western blot analysis also revealed an increased amount of cell cycle inhibitor p21 and decreased amount of cyclinB1 promotes G2/M progression at 24 h after YS110 treatment. These alterations may be compatible with the retarded G2/M transition.

To determine the upstream regulator of the altered cdc25C phosphorylation state caused by YS110, we investigated several molecules known to regulate cdc25C. 24 h after YS110 treatment, activated phosphorylation levels of p38 MAPK (Thr180/Thy182) and ERK 1/2 (Thr202/Tyr204) were elevated. Inhibitor assays indicated that YS110 treatment activated the ERK signal pathway, but not the p38MAPK pathway, which induced G2/M delay.

MAPK activation results in many different biological responses, including proliferation, differentiation, and cell death. Although ERK1/2 activation is associated with cell survival and proliferation, a number of studies have shown that activation of ERK1/2 can mediate cell cycle arrest and cell death depending on the stimuli and cell types involved [17]. A number of anti-cancer reagents and an anti-cancer antibodies have been reported to induce G2/M cell cycle arrest and/or apoptosis mediated by ERK1/2 activation [18, 19]. These reagents, like YS110, cause G2/M cell cycle delay by inhibiting phosphorylation of cdc2 and cdc25C and by activating phosphorylation of ERK1/2. The G2/M delay caused by these drugs is antagonized by MEK inhibitors. Among monoclonal antibodies that have been investigated for their anti-cancer characteristics, the anti-CD40 antibody has been reported to cause apoptosis of diffuse large B-cell lymphoma cell lines with ERK1/2 activation through CD40 signaling [20]. In these reported cases, as well as our findings of YS110-CD26 interaction, the mechanisms underlying G2/M delay or apoptosis through ERK1/2 activation is still unknown. As for G1/S cell cycle regulation, a report has indicated that HER2 signaling related to trastuzumab treatment had effects on CDC25A protein stability [21]. The involvement of cell cycle regulators on the effect of anti-tumor antibodies should be further investigated.

Previously, we reported that YS110 induces intra-nuclear transportation of CD26. When bound to YS110, CD26 is translocated to the nucleus via caveolin-dependent endocytosis. This translocation suppresses transcription of the POLR2A gene, which encodes a large subunit of RNA polymerase, in MM cell lines [22, 23]. The relationship between POLR2A suppression and G2/M cell cycle delay also requires further investigation.

The NCI-H2452 cell line is derived from the epithelioid type of MM and its growth rate is lower than the sarcomatoid type of MM, the JMN cell line [24]. Profiles of cell cycling for synchronization assay in S-phase by thymidine block are available for NCI-H2452 cells but not for JMN cells. NCI-H2452 cells have no tumorigenicity in immunodeficient mice but JMN cells form subcutaneous tumors or diffuse and spread into the thorax [11, 22, and 23]; therefore, after YS110 treatment, we examined the in vitro cell cycle using NCI-H2452 cells and in the in vivo xenograft model using JMN cells.

Pemetrexed (PMX) induced augmented CD26 expression in both NCI-H2452 cells and JMN cells rapidly (Fig. 5b); this induction of cell surface CD26 on MM cells may be useful for anti-CD26 MoAb therapy against MM. The combination of YS110 and PMX therapy against xenografted MM tumors was applied using JMN cell transplanted immunodeficient mice. As a result, anti-tumor effects were significantly shown in xenografted tumors with YS110 and PMX combined treatment compared to tumors with single treatment of YS110 or PMX, which was accompanied by a significantly reduced MIB-1 index. The combined treatment with YS110 and PMX showed a tendency to retard both G1/S and G2/M transition; however, significant differences of G1/S or G2/M proportion were not seen between YS110 plus PMX treatment and YS110 or PMX single treatment (data not shown).

There have been many anti-cancer monoclonal antibodies (MoAbs) developed and their anti-cancer mechanisms are highly variable. Investigations into each molecular mechanism of an anti-cancer MoAb are significant because it is valuable for the optimization of antibody therapies against cancer and contributes to elucidating the mechanisms of oncogenesis and cancer proliferation. This is the first report of a novel anti-proliferative mechanism of a humanized anti-CD26 monoclonal antibody YS110 causing G2/M cell cycle delay through ERK1/2 phosphorylation and identifying PMX as a CD26 inducer.

## Conclusions

Humanized anti-CD26 monoclonal antibody YS110 suppressed proliferation of CD26 positive MM cell lines through a novel mechanism causing G2/M cell cycle delay through ERK1/2 phosphorylation. Anti-tumor agent PMX was identified as a CD26 inducer.

## Methods

### Reagents and antibodies

The humanized anti-CD26 antibody YS110 was constructed from the anti-CD26 mouse monoclonal antibody 14D10 coding sequence as previously described [11] and normal human IgG1 (Southern Biotech, Birmingham, AL) was used as a control. Rabbit monoclonal antibody to cyclinB1, p21cip/waf1, cdc2, phospho-cdc2 (Tyr15), cdc25c, phospho-cdc25c (Ser216), Erk1/2, phospho-Erk1/2(Thr202/Tyr204), p38MAPK, and phosphor-p38MAPK (Thr180/Tyr182) were from Cell Signaling Technology Inc. (Danvers, MA) and the mouse monoclonal antibody against β-actin or Glyceraldehyde 3-phosphate dehydrogenase (GAPDH) was from DAKO (Glostrup, Denmark). The goat anti-CD26 polyclonal antibody and MEK 1/2 inhibitor U0126 was from Cell Signaling Technology Inc. (Danvers, MA).

### Cell culture

NCI-H2452, NCI-H28 and JMN, CD26-positive cell lines established from malignant mesothelioma, were kind gifts from Dr. Chikao Morimoto (Departement of Therapy Development and Innovation for Immune Disorders and Cancers, Juntendo University). Both cell lines were grown in RPMI medium (Sigma-Aldrich, Tokyo, Japan) supplemented with 10 % heat-inactivated fetal bovine serum (FBS), ABPC (100 unit/mL), Streptomycin (100 μg/mL), and 5 % CO_2_ at 37 °C.

### Cell proliferation assay

The effect of YS110 on the proliferation of NCI-H2452 cells was measured using a colorimetric cell proliferation kit WST-1 (Roche Diagnostics, Tokyo, Japan) based on the colorimetric detection of a formazan salt. In brief, 5 × 10^3^ NCI-H2452 cells were seeded in RPMI1640 medium supplemented with 10 % heat inactivated FBS on 96-well plate with or without 2 μg/mL YS110. After 48 h of incubation at 37 °C in 5 % CO_2_, a reading at 450 nm was carried out according to the manufacturer’s instructions. Background absorbance of each sample at 630 nm was subtracted from the readings at 450 nm. The experiment was performed in triplicate and a representative experiment is shown.

### Cell cycle assay and flowcytometry

For the cell cycle study, NCI-H2452 and NCI-H28 cells were synchronized in the S-phase by a repeated thymidine block. In brief, 5 × 10^5^ cells seeded in RPMI1640 medium supplemented with 10 % heat inactivated fetal bovine serum on 10 cm culture dishes were treated with 0.56 mM thymidine for 18 h, released for 10 h by three washes, and then treated again with 0.56 mM thymidine for 15 h. Synchronized cells were then returned to thymidine free medium with or without 2 μg/mL YS110 and incubated for 24 h. Cell cycle profiles were performed by flowcytometry using a procedure for propidium iodide staining of nuclei. Acquisition was performed using an EPICS XL/XL-MCL version 3.0 (Beckman Coulter, Brea, CA, USA) and data were analyzed using Flowjo software (TreeStar, Ashland, OR, USA). The experiment was performed in triplicate and a representative experiment is shown.

### Western blotting

For total cell lysate preparation, NCI-H2452 cells or JMN cells cultured with or without 2 μg/mL YS110 for 24 h were lysed at 4 °C by lysis buffer with phosphatase inhibitors (50 mM Tris–HCl, 150 mM NaCl, 1 % NP-40, 0.25 % deoxycholate, 500 μM NaVO3, 50 mM NaF). For the preparation of nuclear and cytoplasmic extracts, NCI-H2452 cells were processed using NE-PER Nuclear and Cytoplasmic Extraction Reagents (Thermo scientific, Waltham, LA) according to the manufacturer’s instruction. For Western blot analyses, 30 μg of each cell lysate was separated on an SDS–polyacrylamide gel and transferred to a PVDF membrane Hybond-P (GE Healthcare, Little Chalfont, UK). The membranes were blocked in blocking buffer [5 % dry milk and 0.2 % Tween 20 in Tris buffered saline (TBS)] for 2 h at room temperature, and incubated with the primary antibodies in antibody dilution buffer (5 % bovine serum albumin, 0.2 % Tween 20 in TBS) overnight at 4 °C. Dilutions of primary antibodies were 1:200, except anti p21cip/waf1 antibody, which was diluted at 1:100. After three washes, the blots were incubated with secondary antibodies (goat anti-rabbit polyclonal antibody and rabbit anti-mouse polyclonal antibody) diluted 1:1000 with dilution buffer for 1 h at room temperature and developed using the ECL Western Blotting Detection Reagents (GE Healthcare, Little Chalfont, UK). Quantification of relative band densities was performed using standard densitometry scanning techniques using ImageQuant 350 and ImageQuant TL software (GE Healthcare, Little Chalfont, UK). All the experiments performed in triplicate and a representative experiment is shown.

### Xenograft model using human mesothelioma cell lines

NOD/Shi-scid, IL-2 receptor gamma null (NOG) mice were obtained from the Central Institute for Experimental Animals. JMN cells (1 × 10^6^) were implanted subcutaneously in the back flank of NOG mice. Mice were injected intratumorally with control human IgG_1_ (n = 3) or YS110 (n = 3) at doses of 5 mg/kg body weight. JMN cells expressing CD26 were inoculated into the thoracic cavities of NOG mice. Thereafter, mice were intraperitoneally injected with control human IgG_1_ (n = 3) or YS110 (5 μg per injection; n = 3), and/or pemetrexed (PMX, purchased from Eli Lilly; 100 mg/kg body weight) commencing on the day of cancer cell injection. Each antibody was administered three times per week. Mice were then monitored for the development and progression of tumors. Tumor weight was measured by scale. All experiments were approved by the Animal Care and Use Committee of Keio University and were performed in accordance with the institute guidelines.

### Histology and immunohistochemistry

Tumor tissues were fixed in 10 % neutral buffered formalin, embedded in paraffin, and sectioned at a thickness of 5 μm. Sections were paraffin depleted and rehydrated in a graded series of ethanol solutions. For histology, sections were stained with hematoxylin and eosin. For immunohistochemistry, sections were washed with PBS, subjected to antigen retrieval by heating at 100 °C in 0.01 M sodium citrate (pH 6.0) for 10 min, then treated with 3 % H_2_O_2_, before incubation with the following primary antibodies: goat anti-CD26 pAb (AF1180, R&D Systems, Minneapolis, MN) (1:100) and mouse anti-Ki-67 mAb (MIB-1, DAKO Japan) (1:100). Immune complexes were detected by using an ImmPRESS REAGENT KIT (Vector Laboratories, Burlingame, CA) with 3, 3′-diaminobenzidine, and sections were counterstained with hematoxylin

### Statistical analyses

Statistical analyses were assessed using SPSS version 17.0 (SPSS Inc., Chicago, IL). The *p* value, from which statistical significance was assumed, was set to *p* < 0.05.

## Abbreviations

MM: malignant mesothelioma
PMX: pemetrexed
NOG mouse: NOD/Shi-scid, IL-2 receptor gamma null mouse

## References

[1] Sugarbaker DJ (2009). Multimodality management of malignant pleural mesothelioma: introduction. Semin Thorac Cardiovasc Surg.

[2] Krug LM, Pass HI, Rusch VW, Kindler HL, Sugarbaker DJ, Rosenzweig KE, Flores R, Friedberg JS, Pisters K, Monberg M, Obasaju CK, Vogelzang NJ (2009). Multicenter phase II trial of neoadjuvant pemetrexed plus cisplatin followed by extrapleural pneumonectomy and radiation for malignant pleural mesothelioma. J Clin Oncol.

[3] Vogelzang NJ, Rusthoven JJ, Symanowski J, Denham C, Kaukel E, Ruffie P, Gatzemeier U, Boyer M, Emri S, Manegold C, Niyikiza C, Paoletti P (2003). Phase III study of pemetrexed in combination with cisplatin versus cisplatin alone in patients with malignant pleural mesothelioma. J Clin Oncol.

[4] Van Schil PE, Baas P, Gaafar R, Maat AP, Van de Pol M, Hasan B, Klomp HM, Abdelrahman AM, Welch J, van Meerbeeck JP (2010). Trimodality therapy for malignant pleural mesothelioma: results from an EORTC phase II multicentre trial. Eur Respir J.

[5] Greillier L, Marco S, Barlesi F (2011). Targeted therapies in malignant pleural mesothelioma: a review of clinical studies. Anticancer Drugs.

[6] Ohnuma K, Uchiyama M, Yamochi T, Nishibashi K, Hosono O, Takahashi N, Kina S, Tanaka H, Lin X, Dang NH, Morimoto C (2007). Caveolin-1 triggers T-cell activation via CD26 in association with CARMA1. J Biol Chem.

[7] Bauvois B (2004). Transmembrane proteases in cell growth and invasion: new contributors to angiogenesis?. Oncogene.

[8] Aoe K, Amatya VJ, Fujimoto N, Ohnuma K, Hosono O, Hiraki A, Fujii M, Yamada T, Dang NH, Takeshima Y, Inai K, Kishimoto T, Morimoto C (2012). CD26 overexpression is associated with prolonged survival and enhanced chemosensitivity in malignant pleural mesothelioma. Clin Cancer Res.

[9] Amatya VJ, Takeshima Y, Kushitani K, Yamada T, Morimoto C, Inai K (2011). Overexpression of CD26/DPPIV in mesothelioma tissue and mesothelioma cell lines. Oncol Rep.

[10] Antczak C, De Meester I, Bauvois B (2001). Ectopeptidases in pathophysiology. Bio Essays.

[11] Inamoto T, Yamada T, Ohnuma K, Kina S, Takahashi N, Yamochi T, Inamoto S, Katsuoka Y, Hosono O, Tanaka H, Dang NH, Morimoto C (2007). Humanized anti-CD26 monoclonal antibody as a treatment for malignant mesothelioma tumors. Clin Cancer Res.

[12] Le X-F, Pruefer F, Bast RC (2005). HER2-targeting antibodies modulate the cyclin-dependent kinase inhibitor p27Kip1 via multiple signaling pathways. Cell Cycle.

[13] Vincenzi B, Schiavon G, Silletta M, Santini D, Tonini G (2008). The biological properties of cetuximab. Crit Rev Oncol Hematol.

[14] Weiner GJ (2010). Rituximab: mechanism of action. Semin Hematol.

[15] Wu JD, Odman A, Higgins LM, Haugk K, Vessella R, Ludwig DL, Plymate SR (2005). In vivo effects of the human type I insulin-like growth factor receptor antibody A12 on androgen-dependent and androgen-independent xenograft human prostate tumors. Clin Cancer Res.

[16] Takizawa CG, Morgan DO (2000). Control of mitosis by changes in the subcellular location of cyclin-B1-Cdk1 and Cdc25C. Curr Opin Cell Biol.

[17] Mebratu Y, Tesfaigzi Y (2009). How ERK1/2 activation controls cell proliferation and cell death: is subcellular localization the answer?. Cell Cycle.

[18] Zhang Z, Miao L, Lv C, Sun H, Wei S, Wang B, Huang C, Jiao B (2013). Wentilactone B induces G2/M phase arrest and apoptosis via the Ras/Raf/MAPK signaling pathway in human hepatoma SMMC-7721 cells. Cell Death Dis.

[19] Chuang M-J, Wu S-T, Tang S-H, Lai X-M, Lai H-C, Hsu K-H, Sun K-H, Sun G-H, Chang S-Y, Yu D-S, Hsiao P-W, Huang S-M, Cha T-L (2013). The HDAC inhibitor LBH589 induces ERK-dependent prometaphase arrest in prostate cancer via HDAC6 inactivation and down-regulation. PLoS One.

[20] Hollmann CA, Owens T, Nalbantoglu J, Hudson TJ, Sladek R (2006). Constitutive activation of extracellular signal-regulated kinase predisposes diffuse large B-cell lymphoma cell lines to CD40-mediated cell death. Cancer Res.

[21] Brunetto E, Ferrara AM, Rampoldi F, Talarico A, Cin ED, Grassini G, Spagnuolo L, Sassi I, Ferro A, Cuorvo LV, Barbareschi M, Piccinin S, Maestro R, Pecciarini L, Doglioni C, Cangi MG (2013). CDC25A protein stability represents a previously unrecognized target of HER2 signaling in human breast cancer: implication for a potential clinical relevance in trastuzumab treatment. Neoplasia.

[22] Yamada K, Hayashi M, Du W, Ohnuma K, Sakamoto M, Morimoto C, Yamada T (2009). Localization of CD26/DPPIV in nucleus and its nuclear translocation enhanced by anti-CD26 monoclonal antibody with anti-tumor effect. Cancer Cell Int.

[23] Yamada K, Hayashi M, Madokoro H, Nishida H, Du W, Ohnuma K, Sakamoto M, Morimoto C, Yamada T (2013). Nuclear localization of CD26 induced by a humanized monoclonal antibody inhibits tumor cell growth by modulating of POLR2A transcription. PLoS ONE.

[24] Penno MB, Askin FB, Ma H, Carbone M, Vargas MP, Pass HI (1995). High CD44 expression on human mesotheliomas mediates association with hyaluronan. Cancer J Sci Am..

